# A Hybrid Model for Psoriasis Subtype Classification: Integrating Multi Transfer Learning and Hard Voting Ensemble Models †

**DOI:** 10.3390/diagnostics15010055

**Published:** 2024-12-28

**Authors:** İsmail Anıl Avcı, Merve Zirekgür, Barış Karakaya, Betül Demir

**Affiliations:** 1 Department of Electrical-Electronics Engineering, Faculty of Technology, Firat University, 23200 Elazig, Turkey; iaavci@firat.edu.tr; 2 Department of Electrical and Electronics Engineering, Faculty of Engineering and Natural Sciences, Malatya Turgut Ozal University, 44200 Malatya, Turkey; merve.zirekgur@ozal.edu.tr; 3 Department of Electrical-Electronics Engineering, Faculty of Engineering, Firat University, 23200 Elazig, Turkey; 4 Department of Dermatology, Faculty of Medicine, Firat University, 23200 Elazig, Turkey; betuldemir@firat.edu.tr

**Keywords:** dermatological image analysis, ensemble learning, hybrid learning models, psoriasis classification, transfer learning

## Abstract

**Background:** Psoriasis is a chronic, immune-mediated skin disease characterized by lifelong persistence and fluctuating symptoms. The clinical similarities among its subtypes and the diversity of symptoms present challenges in diagnosis. Early diagnosis plays a vital role in preventing the spread of lesions and improving patients’ quality of life. **Methods:** This study proposes a hybrid model combining multiple transfer learning and ensemble learning methods to classify psoriasis subtypes accurately and efficiently. The dataset includes 930 images labeled by expert dermatologists from the Dermatology Clinic of Fırat University Hospital, representing four distinct subtypes: generalized, guttate, plaque, and pustular. Class imbalance was addressed by applying synthetic data augmentation techniques, particularly for the rare subtype. To reduce the influence of nonlesion environmental factors, the images underwent systematic cropping and preprocessing steps, such as Gaussian blur, thresholding, morphological operations, and contour detection. DenseNet-121, EfficientNet-B0, and ResNet-50 transfer learning models were utilized to extract feature vectors, which were then combined to form a unified feature set representing the strengths of each model. The feature set was divided into 80% training and 20% testing subsets and evaluated using a hard voting classifier consisting of logistic regression, random forest, support vector classifier, k-nearest neighbors, and gradient boosting algorithms. **Results:** The proposed hybrid approach achieved 93.14% accuracy, 96.75% precision, and an F1 score of 91.44%, demonstrating superior performance compared to individual transfer learning models. **Conclusions:** This method offers significant potential to enhance the classification of psoriasis subtypes in clinical and real-world settings.

## 1. Introduction

Psoriasis is a chronic inflammatory skin disease that leads to lesions on the skin surface by causing skin cells to regenerate much faster than normal. While healthy skin cells regenerate approximately once a month, psoriatic skin cells regenerate within a few days. This abnormally rapid regeneration results in the accumulation of cells on the skin, causing itchy, inflamed, red lesions covered with thick, silvery scales [1].

Psoriasis manifests in various subtypes, each presenting different symptoms on distinct areas of the skin. The most common subtype is plaque psoriasis, also known as psoriasis vulgaris, which affects 80–90% of patients. It is characterized by red, inflamed, and chronically thickened skin [2]. Guttate psoriasis, another subtype, is commonly observed in children and young adults and is characterized by small, drop-shaped lesions. It tends to spread over large areas of the body [3]. Pustular psoriasis is a rare but severe form of the disease, affecting approximately 1–2% of patients. It is distinguished by pus-filled white blisters (pustules) on the skin and often presents with systemic symptoms, such as headache, muscle pain, and high fever. Unlike other psoriasis subtypes, pustular psoriasis may require urgent medical intervention due to its severe manifestations [2].

According to the World Health Organization (WHO), psoriasis affects approximately 2–3% of the global population, with prevalence rates varying across regions. In highly developed regions, such as Northern Europe and North America, the prevalence reaches as high as 4–5%, while in regions like Asia and Africa, it remains around 0.5–1% [4]. This disparity is attributed to the advanced healthcare systems, efficient data utilization, and higher diagnostic accuracy in developed countries. In contrast, factors such as limited access to healthcare services and low disease awareness in less developed regions contribute to the underdiagnosis of psoriasis in these areas.

Dermatologists typically diagnose psoriasis by noninvasively observing skin lesions. However, in some cases, invasive methods, such as biopsies, may be required to confirm the diagnosis or differentiate psoriasis from other skin conditions. Artificial intelligence (AI) techniques offer the potential to achieve highly accurate diagnoses without the need for invasive procedures.

The performance of AI models, however, depends heavily on the availability of well-labeled and high-quality datasets. Such datasets enable the model to accurately learn the characteristics of lesions and distinguish subtle differences between subtypes, thereby enhancing the classification performance [5].

Transfer learning (TL) techniques represent a type of deep learning (DL) that enables the application of model weights and features obtained from training on millions of images for various tasks to a new task. This approach provides a practical solution in scenarios where access to sufficient labeled data is limited or unavailable, enabling high accuracy and rapid predictions. In skin diseases like psoriasis, where rare subtypes often have restricted data availability, the application of TL effectively eliminates this disadvantage. Each TL model offers a unique architecture, capturing distinct features from images to enhance the classification performance [6].

An ensemble learning (EL) classifier combines multiple machine learning (ML) techniques to create a more robust predictive model. By integrating the strengths of different models, EL aims to achieve better predictions, while mitigating the limitations and biases associated with a single model. EL employs various strategies for combining ML techniques, with one of the most commonly used being the hard voting classifier (HVC). This method collects the independent predictions from each selected ML technique and determines the final prediction based on majority voting, where the outcome with the highest votes is chosen as the result [7].

In this study, we combined TL’s strong feature extraction and generalization capabilities on limited datasets with EL’s ability to integrate the best aspects of various machine learning techniques. This approach introduces a hybrid method that leverages both TL and EL techniques, proposing a highly generalizable model for the diagnosis of psoriasis subtypes. For this purpose, we utilized a unique dataset consisting of images of psoriasis patients treated at the Dermatology Clinic of Firat University Hospital. The dataset, labeled by expert dermatologists, comprises 930 images representing four psoriasis subtypes: generalized, guttate, plaque, and pustular psoriasis.

**Figure 1:**
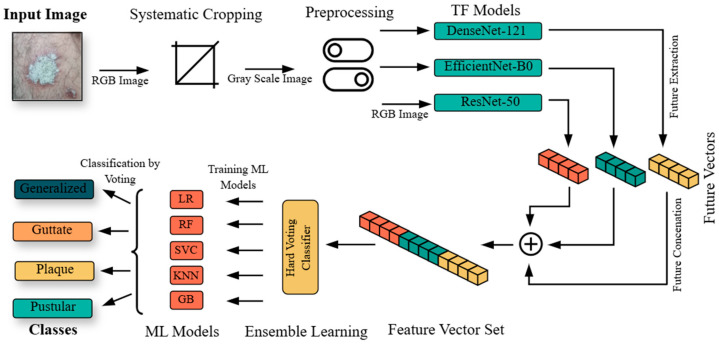
An overview of the proposed approach for integrating multi-TL and EL models.

The proposed approach in this study is illustrated in Figure 1. The preprocessing of the dataset and implementation steps of the approach are as follows:

- Initially, nonlesion areas were systematically cropped, and noise was reduced using Gaussian blur. Brightness equalization, morphological operations, contour detection, and re-cropping steps were applied to optimize the dataset for analysis;
- The minority class in the dataset, pustular, was synthetically augmented to prevent models from exhibiting overfitting;
- The dataset was divided into 80% training and 20% testing. The training dataset was further split into 80% training and 20% validation subsets;
- DenseNet-121, EfficientNet-B0, and ResNet-50 models were trained and evaluated using this dataset;
- The feature vectors obtained from the final layers of the TL models were saved in h5. format. These vectors were combined to form a unified feature vector set;
- The combined feature set was divided into 80% training and 20% testing subsets;
- A hard voting classifier, incorporating logistic regression (LR), random forest (RF), support vector classifier (SVC), k-nearest neighbors (KNN), and gradient boosting (GB) algorithms, was used to retrain and evaluate the feature vector set.

The classification performed by combining multiple TL models and EL achieved 93.14% accuracy, 96.75% precision, and an F1 score of 91.44%, surpassing the individual performance of the three TL models used.

In the subsequent sections of the article, Section 2 reviews previous studies in the literature on the diagnosis of psoriasis. Section 3 introduces the dataset used in this study and provides detailed explanations of the preprocessing steps. Section 4 and Section 5 present the methodology and analysis of the results, respectively. Section 6 and Section 7 are dedicated to the discussion and conclusion.

## 2. Related Works

Psoriasis is a skin disease with multiple subtypes, typically diagnosed through the direct observation of lesions. TL and various ensemble classifier techniques are widely used in the diagnosis and classification of the disease. In the literature, numerous machine learning methods and their combinations are frequently compared for the diagnosis of psoriasis. The presence of some rare subtypes often leads to imbalanced datasets, necessitating the use of data balancing techniques to address this issue effectively.

Syu et al. proposed a computer-aided diagnosis (CAD) system for psoriasis detection based on deep neural networks (DNN). For this purpose, they utilized a dataset consisting of 5700 images of normal and psoriatic skin. The developed model achieved 91% accuracy on the test dataset and over 95% accuracy on the training dataset [8].

Pal et al. utilized the simple linear iterative clustering (SLIC) algorithm for the segmentation of psoriasis biopsy images and the deep convolutional neural network (DCNN) for classification. Their study, conducted on 90 images of psoriasis biopsies, included comparisons with KNN, SVM, and RF. The fully convolutional network (FCN) model achieved a ratio of correct pixel classification (RCPC) of 0.8801 ± 0.0780, a Jaccard coefficient (JC) of 0.7717 ± 0.1476 for the dermis, and 0.7710 ± 0.1267 for the epidermis [9].

Amin and Farooq employed TL and a 2D convolutional neural network (CNN) model to work with an 815-image dataset from the DermNetNZ database. The dataset included images of normal skin, psoriasis, and psoriasis-like skin conditions (e.g., eczema, lichen planus, pityriasis, tinea corporis). In classifications conducted with ResNet-50, DenseNet-121, InceptionV3, and ResNeXt101 models, the highest performance was achieved with the ResNeXt101 model, with 94% accuracy in binary classification and 79% accuracy in three-class classification [10].

Aijaz et al. used TL and long short-term memory (LSTM) models to diagnose five types of psoriasis (guttate, pustular, plaque, inverse, and erythrodermic) and distinguish them from normal skin. They trained the VGG19 TL model and a custom LSTM model using 301 psoriasis images from the DermNet dataset and 172 normal skin images from the BFL NTU dataset. The study achieved 84.2% accuracy with the TL algorithm and 72.3% accuracy with the LSTM model [11].

Goswami et al. employed a CNN-based classifier with an intraclass approach to classify psoriasis subtypes with similar characteristics. They used a dataset consisting of six classes: guttate, pustular, plaque, inverse, erythrodermic psoriasis subtypes, and normal skin, obtained from the DermNet database. The study trained SqueezeNet, GoogleNet, and ResNet-18 TL models in MATLAB and compared their results. The highest performance was achieved with the ResNet-18 model, reaching 93.62% accuracy [12].

Arunkumar and Jayanna developed a MobileNet-based CAD system to estimate the severity of psoriasis. They utilized a dataset comprising 5951 images divided into three classes: mild, moderate, and severe. The classification was performed using depth-wise convolution and the ReLU activation function. The model, optimized for low-power and fast devices, achieved 90% accuracy and an average F1 score of 0.94 [13].

Yaseliani et al. developed a DL-based decision and diagnostic support system (D&DSS) for psoriasis classification and treatment recommendation. This system utilized residual network models, including ResNet-50V2, ResNet-101V2, and ResNet-152V2. A multicriteria decision making (MCDM) technique was employed to create a treatment recommendation system integrated into the diagnostic algorithm. The study used a dataset from the DermNetNZ database, comprising 2100 images (813 psoriasis images and 1287 non-psoriasis images) covering seven psoriasis subtypes and other skin conditions. Initially, the images were classified into psoriasis and non-psoriasis categories. Psoriatic images were then further classified into seven specific subtypes. The ensemble model demonstrated the best performance, achieving 93.29% accuracy and an F1 score of 88.72% [14].

Nieniewski et al. [15] aimed to distinguish psoriasis from other skin diseases using transfer learning techniques. They worked with a dataset comprising 3570 images collected from 75 psoriasis patients and 75 non-psoriasis patients treated at the Medical University of Lodz in Poland. A total of 1280 features extracted from the convolutional layers of the VGG16 model were classified using support vector machines (SVM) with a radial basis function (RBF) kernel. Majority voting achieved 80.08% accuracy, 82.58% precision, and 85.33% recall.

Moon et al. [16] classified psoriasis into severity levels (healthy, mild, moderate, severe, very severe) using a multiscale deformable attention module (MS-DAM) with images obtained from 44 Korean patients. The dataset consisted of 640 images for training and 152 images for testing. Data augmentation techniques, including CutMix and geometric transformations, were applied. MS-DAM was integrated into EfficientNet-B1 and RegNetY models, achieving an F1 score of 0.93.

## 3. Materials and Methods

The section describes the dataset and preprocessing steps of the study to eliminate the influence of nonlesion environmental factors and noise in the dataset.

### 3.1. Dataset and Data Preprocessing

In this study, a unique dataset obtained from 172 psoriasis patients at the Dermatology Outpatient Clinic of Fırat University Hospital, Elazig, Turkey was utilized. The dataset comprised four classes, including 117 generalized, 159 guttate, 26 pustular, and 628 plaque images, for a total of 930 images. To enhance the representativeness of the dataset, images of different lesion areas from some patients were captured from various angles.

Images from 102 male and 70 female patients were collected using phone cameras in an environment close to their daily living conditions. This approach enabled the analysis of the images captured in a natural setting within the study.

**Figure 2:**
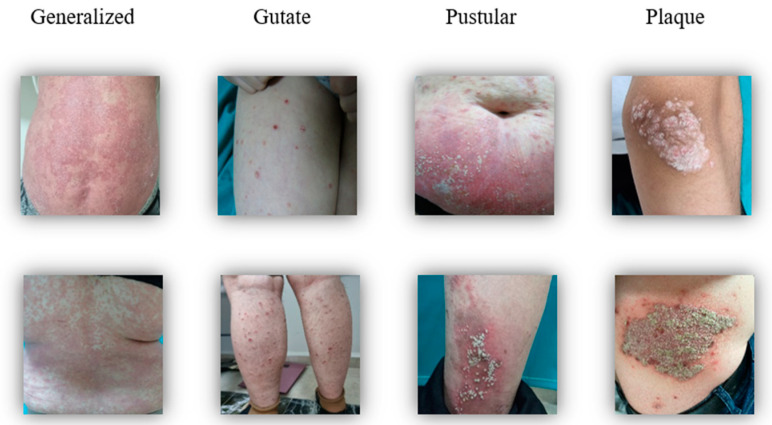
Sample images of psoriasis classes in the dataset.

**Figure 3:**
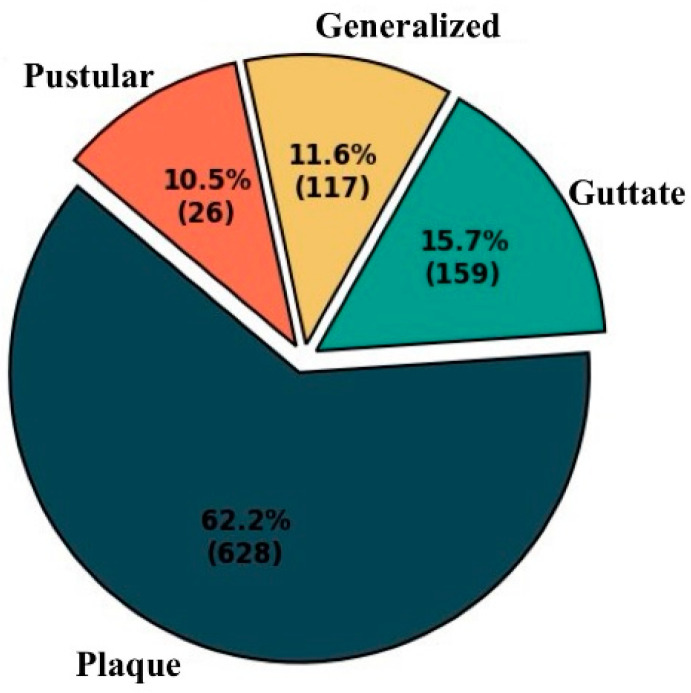
Class distribution of the dataset.

All images were stored in high-resolution JPEG format, ensuring consistency across varying dimensions. This format ensured that the images could be used during analysis without any loss of quality. Examples of the classes in the dataset are presented in Figure 2, while the distribution of patients across the classes is shown in Figure 3.

The success of AI models is directly related to the quality of the dataset used. Therefore, ensuring that all classes in the dataset are adequately represented and well-processed will directly enhance model performance [5]. To achieve optimal model performance, various preprocessing and class balancing techniques were applied to ensure high data quality. This section provides a detailed explanation of the data preprocessing techniques employed in the study.

#### 3.1.1. Preparation and Noise Reduction

The images in the dataset included not only the lesions of interest but also various environmental factors, such as the shooting environment and nonlesion body parts of the patients. These factors complicate image analysis, hinder focus on the lesion area, and may negatively impact model performance. Such external elements should be carefully addressed during image processing and analysis stages and filtered out as much as possible. Accordingly, areas outside the lesions were systematically cropped to isolate the target region for analysis.

The grayscale conversion process was applied as a fundamental image preprocessing step to reduce color noise and improve the quality of analysis [17]. Working with grayscale images during various preprocessing steps, such as contour detection and noise reduction, eliminates the need for separate and complex operations for each color channel. This approach allows filters to perform more effectively on a single intensity level [18].

The Gaussian blur filter was applied to smooth out random brightness variations in images captured under various lighting conditions and with cameras of differing resolutions. This process enhances the visual quality for image processing applications by producing results that are closer to human visual perception [19].

#### 3.1.2. Thresholding and Morphological Operations

At this stage of the study, brightness equalization and morphological operations were performed to make the lesions clearer and easier for the model to identify. These processes create sharper and smoother boundaries around the lesions and isolate them entirely from the background by separating pixels at the determined brightness threshold into two levels.

After the thresholding process, morphological operations were applied to remove small remaining artifacts and create more distinct boundaries. Through erosion, white areas were reduced to eliminate small and isolated artifacts, removing regions other than prominent and significant areas in the image. To prevent the loss of fine details during erosion, dilation was subsequently applied to expand white areas, enhancing the boundaries of the lesions.

#### 3.1.3. Contour Detection and Cropping

Contour extraction involves identifying the outer boundaries of simplified binary images created through thresholding and morphological operations. In binary images, white pixels represent the main object, while black pixels define the background. By excluding internal details and preserving key boundary points, the process aims to optimize memory usage. After identifying the outermost points of the contour, which are defined as the structure covering the largest area and representing the main object, lesions were cropped and isolated along these points. As a result, the dataset became clearer and more focused, preventing the analysis from being influenced by environmental factors and noise.

To improve the preprocessing step, the images, which were converted to two channels for processing, were restored to their original color format after these operations. This ensures that detailed analysis can be performed during model training without losing color information. Each image was then resized to 224 × 224 × 3 dimensions for consistency in the model input.

**Figure 4:**
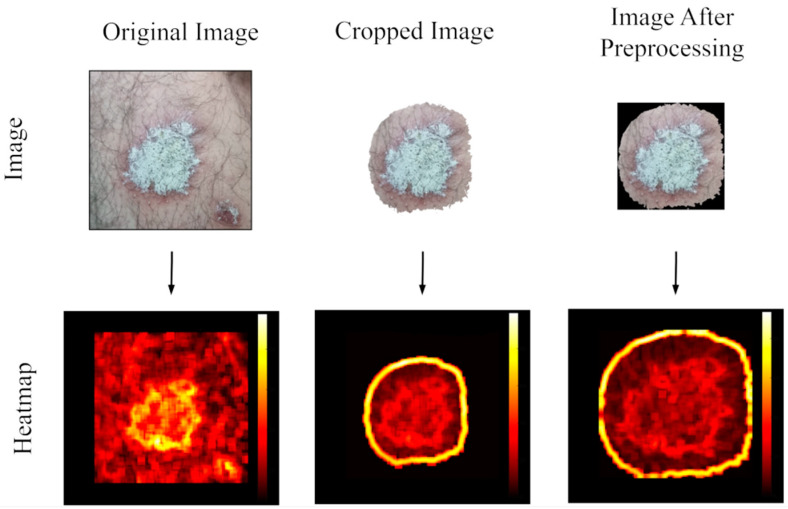
The impact of data preprocessing steps on the image: heat maps of original, cropped, and fully preprocessed images.

Figure 4 illustrates the effects of the image processing steps in a comparative manner. In the original image, unnecessary details and significant texture variations were observed around the skin area containing the lesion. At this stage, a wide heat distribution and relatively high pixel intensity are noticeable. In the cropped image, the removal of environmental redundancies focuses attention exclusively on the lesion region, clearly revealing the textural differences between the central and peripheral areas. In the final image, processed through the preprocessing steps, texture variations are balanced, the lesion center appears more homogeneous, and peripheral textures are more defined. This process ensures greater stability in the lesion center and allows for a clearer definition of the surrounding tissues.

### 3.2. Data Augmentation

For AI models to effectively learn complex patterns, the dataset needs to be sufficiently large [20]. However, imbalanced class distributions can lead to the dominance of the majority class over the minority class in the model’s decision making process [21]. Among the various methods used to address this issue, the most common approach is generating synthetic data for the minority class to increase its representation [22].

The study’s dataset consists of 930 psoriasis images, including 117 generalized, 159 guttate, 628 plaque, and 26 pustular cases. The primary reason for this imbalance is that pustular psoriasis is one of the rarest types globally [23].

Data augmentation increases the number of images in the pustular class synthetically. However, as data augmentation can elevate the risk of overfitting [24], not all images in the class were augmented. Instead, 16 images were selected, and each was augmented five times, resulting in a total of 106 images. Table 1 summarizes the specific steps and parameters used in data preprocessing and data augmentation.

## 4. Proposed Model

The proposed method in this study aims to classify four different subtypes of psoriasis using a hybrid approach that integrates multi-TL and EL techniques. For this purpose, a dataset consisting of images from 930 patients treated at the Dermatology Outpatient Clinic of Fırat University Hospital was used. The dataset includes four psoriasis subtypes: generalized, guttate, plaque, and pustular.

After preprocessing steps, the images were used to train three different TL models: DenseNet-121 [25], EfficientNet-B0 [6], and ResNet-50 [26], initialized with pretrained weights from ImageNet [27]. The performance of these models was evaluated using performance metrics, and only the feature extraction layers were utilized during training. The final layer weights derived from the features extracted by the TL models were combined to create a new set of weights that integrated the strengths of each model. Using this robust and enhanced set of weights, reclassification was performed with an ensemble classifier, the HVC.

The classifier, consisting of five different ML models, LR, RF, SVC, KNN, and GB, determined the winning class through a majority voting method.

For feature extraction with TL models, 20% of the dataset was divided as the test set, while the remaining 80% was further split into 80% for training and 20% for validation. The set of feature vectors was then divided into 80% for training and 20% for testing for use in the EL phase.

### 4.1. Callback Functions

For during the training of TL models, various strategies and callback functions were employed to optimize the training process and enhance the model’s generalization capability.

The ModelCheckpoint function was configured to monitor the validation accuracy and save the model at the point where it demonstrated the best generalization ability during training. Another potential issue that could lead to overfitting is when the model continues training without improving on the validation set. To prevent this, early stopping was implemented, halting training if no changes were observed in the validation accuracy. This approach not only avoids overfitting but also prevents unnecessary consumption of time and resources.

The ReduceLROnPlateau callback function was used to monitor the model’s validation performance. When no significant improvement was observed, adjustments were made to the learning rate. By reducing the learning rate, the model’s weights were updated with smaller steps, allowing for finer optimization. As training progresses, a high learning rate can hinder the model from reaching the optimal minimum [28]. The callback function parameters used for the three TL models are presented in Table 2.

### 4.2. TL Models and Fine Tuning

TL enables the use of models trained in source domains to leverage their existing knowledge for target domains, particularly in problems where access to sufficiently large and labeled datasets is limited [29]. Even when there are differences in data distribution and feature space between the source and target domains, TL models can still be applied, significantly broadening their scope of application [30].

In this study, deep neural network architectures known for their efficiency and unique designs were employed. These include DenseNet-121 [25], which provides high accuracy with a low number of parameters and connects each layer to all previous layers; EfficientNet-B0 [31], which optimizes the width, depth, and resolution scales using compound scaling; and ResNet-50 [32], which prevents the vanishing gradient problem through the use of residual connections.

TF models pretrained on the ImageNet dataset, which contains over 14 million images and more than 20,000 classes, were utilized solely for feature extraction by leveraging their final classification layers. Additionally, fine-tuning was performed to ensure optimal performance of the TF models. During this process, the trainable parameters of the models were adjusted using the dataset to refine their performance.

The DenseNet-121 model is composed of four dense blocks consisting of convolutional layers, followed by transition layers. The input layer includes a 7 × 7 convolution layer and a 3 × 3 max pooling layer. While the dense blocks contain a specific number of convolutional layers, the transition layers include 1 × 1 convolutions and 2 × 2 average pooling layers. All layers in the model are directly connected [33], allowing information to be transferred without redundant relearning and preventing the vanishing gradient problem. The DenseNet-121 architecture consists of a total of 7,041,604 parameters, of which 6,957,956 are trainable.

EfficientNet-B0 enhances efficiency by scaling the model’s depth, width, and resolution using the compound scaling technique [6]. The input layer incorporates a 3 × 3 convolution, batch normalization, and the swish activation function. Feature maps are extracted through optimized MbConvolution structures, which include compression and expansion operations. The model consists of seven main blocks, with the number of output channels in each block determined by the expansion factor. The EfficientNet-B0 architecture used in this study comprises a total of 4,054,695 parameters, of which 4,012,672 are trainable.

ResNet-50 is a DL model consisting of 50 layers and is part of the residual networks family. The input layers include a 7 × 7 convolution, batch normalization, ReLU activation, and 3 × 3 max pooling. These layers are followed by residual blocks, each containing bottleneck residual blocks composed of 1 × 1, 3 × 3, and another 1 × 1 convolutional layers. Skip connections, which directly pass the input to the output, simplify learning [34]. The ResNet-50 architecture used in this study comprises a total of 23,595,908 parameters, of which 23,542,788 are trainable.

**Figure 5:**
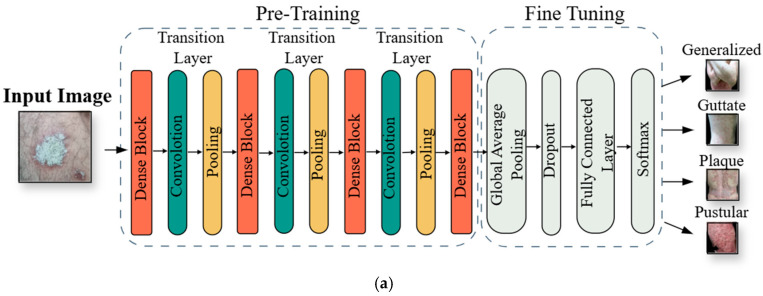

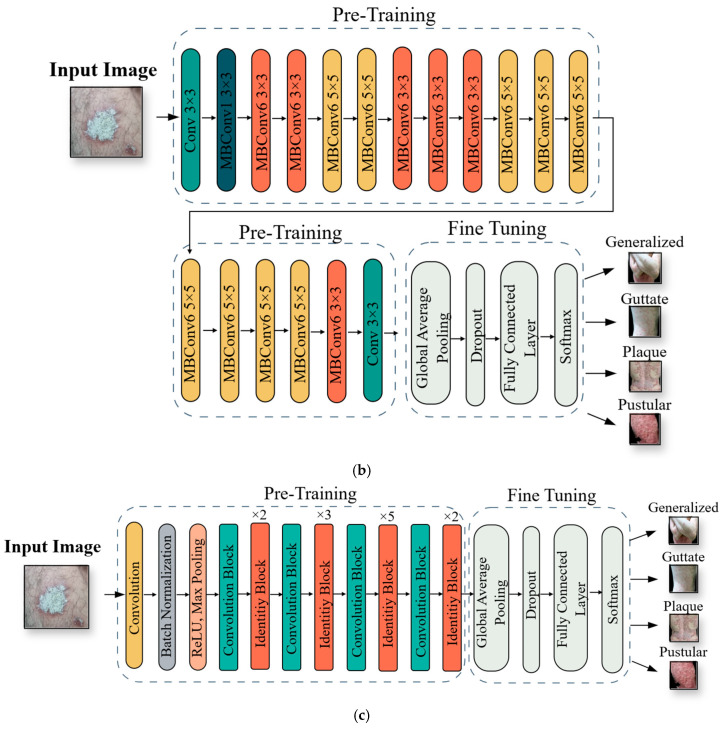
The architecture of TL models: (**a**) DenseNet-121, (**b**) EfficientNet-B0, (**c**) ResNet-50.

The input size of each of the three transfer learning models was 224 × 224 × 3. To prevent overfitting and reduce the number of model parameters, a GlobalAveragePooling2D (GAP) layer was utilized as a fine-tuning layer in all models [35]. Additionally, to further mitigate the risk of overfitting, a dropout layer with a rate of 20% was incorporated [36]. The models were trained for 100 epochs with a learning rate (lr) of 0.0001. Following the fully connected layer, the softmax activation function was applied for multiclass classification, optimizing the classification performance of the model [24]. The architectures of the DenseNet-121, EfficientNet-B0, and ResNet-50 models used in the study are illustrated in Figure 5.

### 4.3. Creation of the Feature Vector Set

Each of the transfer learning (TL) models loaded with pretrained weights generated separate feature vectors for each image. Since the feature vectors were extracted from the same image, they were concatenated side by side to form a single long vector through the concatenation process.

With this method, each image was initially converted into a NumPy array. The images were scaled and normalized to match the inputs of the TL models. The features extracted by each model were converted into one-dimensional arrays and saved in h5. file format for subsequent concatenation.

In this way, each TL model extracted different types of features from the same image. By combining these feature vectors, the strengths of each model were utilized, resulting in a more comprehensive and representative feature map. EfficientNet-B0, with its efficiency-focused design, provided smaller and optimized weights. ResNet-50, with its ability to capture complex structures and fine details, focused on extracting low- and mid-level features, such as edges and textures. Meanwhile, DenseNet-121, with its architecture allowing extensive information sharing through connections between all layers, aimed to extract high-level features.

### 4.4. EL Model

In this study, the HVC, a prediction tool that combines different models to utilize the strengths of various classification techniques, was employed. HVC is commonly applied alongside methods, such as bagging and stacking [37]. The prediction from each model was treated as a vote, and the output of the ensemble classifier was determined by the class with the highest number of votes. In this approach, attention must be given to the relationship between the number of classes and models. In multiclass scenarios, as in this study, the likelihood of models assigning votes to different classes increases, necessitating a sufficient number of models to ensure that a majority vote can converge on a single class.

The HVC model used in this study incorporated five different ML models: LR, RF, SVC, KNN, and GB. The feature vector set was divided into 80% training and 20% testing. Each model within the HVC was trained on the training data. Subsequently, predictions were made on the test feature set, and the final class prediction was determined using the majority voting principle.

## 5. Results and Performance Analyses

The study utilized a dataset of 930 images from four different types of psoriasis: generalized, guttate, pustular, and plaque. The images in the dataset contained environmental factors and noise unrelated to the examined lesions. Preprocessing steps, such as systematic cropping, resizing, Gaussian filtering, and morphological operations, were applied to eliminate these external factors. The images were converted to a two-channel format for ease of preprocessing and then restored to a three-channel format before training. The input image size for the models used in the study was 224 × 224 × 3. To address the dataset imbalance, the minority class, pustular, was augmented using synthetic methods. The dataset was split into 80% training and 20% testing, while the training set was further divided into 80% training and 20% validation subsets.

In this study, three different TL techniques were utilized: DenseNet-121, EfficientNet-B0, and ResNet-50. The final layers of these three TL models were fine-tuned, and after training them with the dataset, the final layer weights were saved. Using these weights, feature vectors were generated for each image in the dataset with each TL model. The combination of these feature vectors formed the feature vector set.

The feature vector set was divided into 80% training and 20% testing. Classification was performed in EL using the HVC with models, such as LR, RF, SVC, KNN, and GB. The performance of the EL and TL models was evaluated using performance metrics including precision, recall, F1 score, specificity, and accuracy.

### 5.1. Performance of TL Models

**Figure 6:**
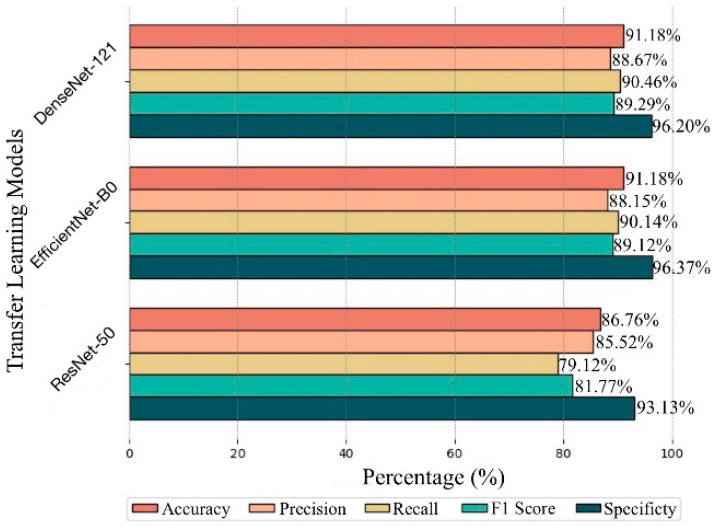
Comparison of performance metrics across TL models.

The performance of the DenseNet-121, EfficientNet-B0, and ResNet-50 TL models was evaluated using the metrics accuracy, precision, recall, F1 score, and specificity. The highest accuracy (91.18%) was achieved by both DenseNet-121 and EfficientNet-B0, with these two models exhibiting similar performance across all other metrics. The DenseNet-121 model demonstrated balanced success with 88.67% precision, 90.46% recall, 89.29% F1 score, and 96.20% specificity. The EfficientNet-B0 model slightly outperformed DenseNet-121 in specificity with a value of 96.37%. In contrast, the ResNet-50 model showed the lowest performance, with 86.76% accuracy, 85.52% precision, 79.12% recall, 81.77% F1 score, and 93.13% specificity. The performance metrics of the three TL models are presented in Figure 6.

**Figure 7:**
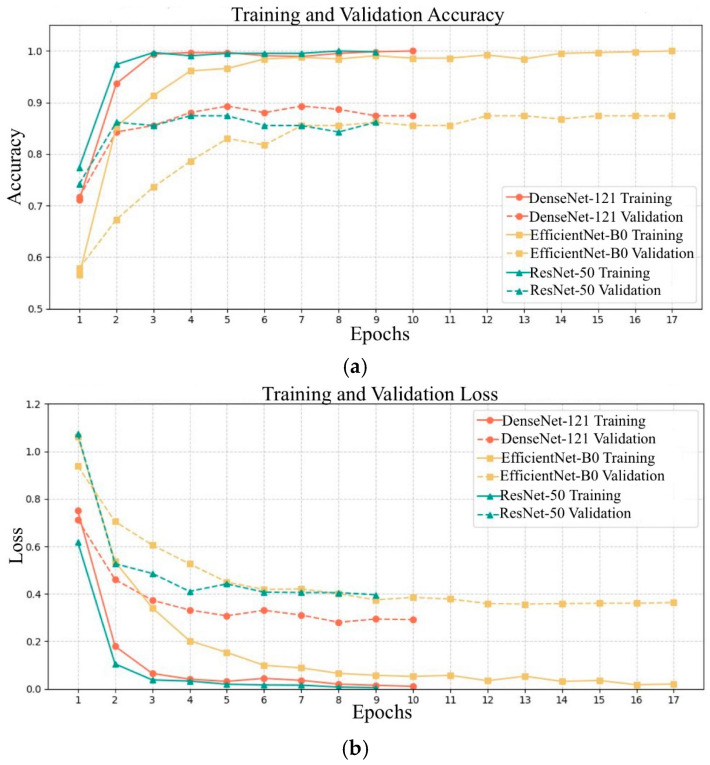
The training and validation performances of the TL models: (**a**) training and validation accuracy, (**b**) training and validation loss.

The training and validation loss and accuracy graphs for the models are presented in Figure 7. The ResNet-50 model demonstrated the shortest training time due to the effect of the early stopping callback function. Conversely, the DenseNet-121 model had the longest training time and exhibited the most consistent performance in terms of both validation and loss metrics compared to the other two models. While EfficientNet-B0 achieved accuracy levels similar to DenseNet-121, its performance was less stable due to fluctuations in validation loss. Although ResNet-50 had the shortest training time, the gap between its training and validation metrics was more pronounced compared to the other models, indicating a higher tendency towards overfitting.

### 5.2. Performance of HVC

**Figure 8:**
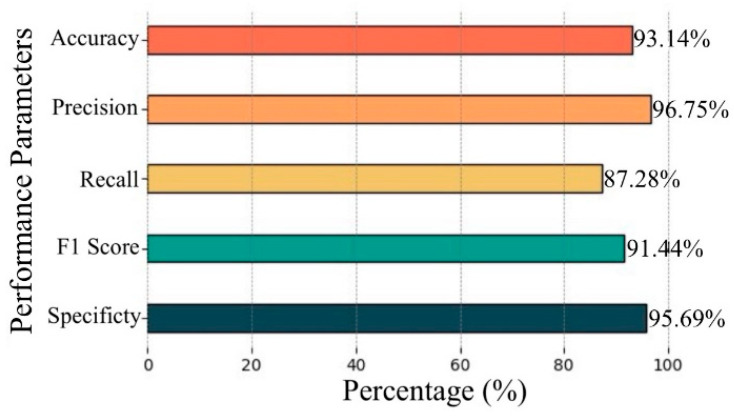
Performance metrics of the HVC model.

In this study, the HVC model demonstrated the highest performance across a broad range of metrics. Figure 8 illustrates the model’s performance metrics. The model achieved 93.14% accuracy, 96.75% precision, 87.28% recall, 91.44% F1 score, and 95.69% specificity. These results indicate that the HVC model provides a balanced performance between accuracy and specificity, while also delivering strong precision and recall.

**Figure 9:**
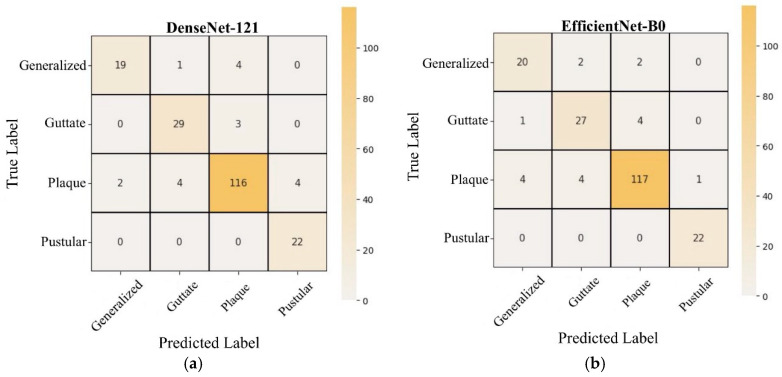

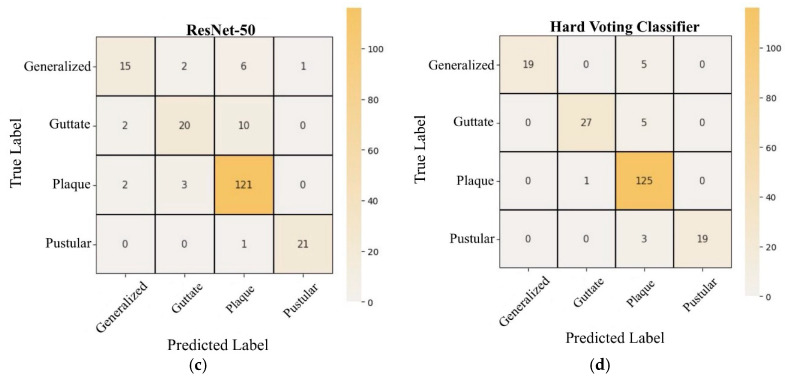
Confusion matrices for each model: (**a**) DenseNet-121, (**b**) EfficientNet-B0, (**c**) ResNet-50, (**d**) HVC.

**Figure 10:**
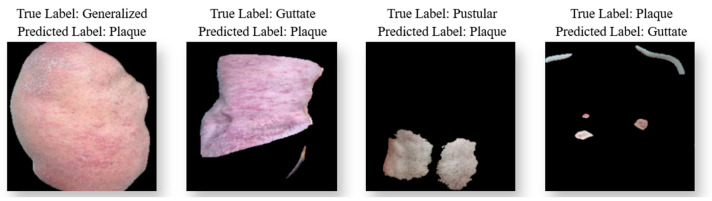
Images for misclassified samples in the HVC model. The Predicted Label indicates the model’s prediction, while the True Label represents the actual class value.

The confusion matrices for the test data of the TL and HVC models used in the study are presented in Figure 9. Additionally, Figure 10 displays examples of images that were misclassified by the HVC model. The dataset images typically display only a limited lesion area. Although lesions in subtypes like generalized are spread across larger body areas, the images showing restricted regions were misclassified by the model as the plaque subtype. This is likely due to the fact that plaque lesions are generally confined to smaller areas. Additionally, some images suffered from quality issues, such as loss of clarity in lesions and insufficient image resolution, which also contributed to misclassifications.

In order to demonstrate the effectiveness of the data augmentation process for the Pustular class, the training results without data augmentation are provided in Table 3.

Without data augmentation, the DenseNet-121, EfficientNet-B0, ResNet-50, and HVC models achieved accuracy rates of 83%, 86%, 86%, and 89%, respectively. The data augmentation process significantly enhanced model performance by addressing imbalanced class distributions. The model-based performance metrics for each class after data augmentation are also presented in Table 4.

**Figure 11:**
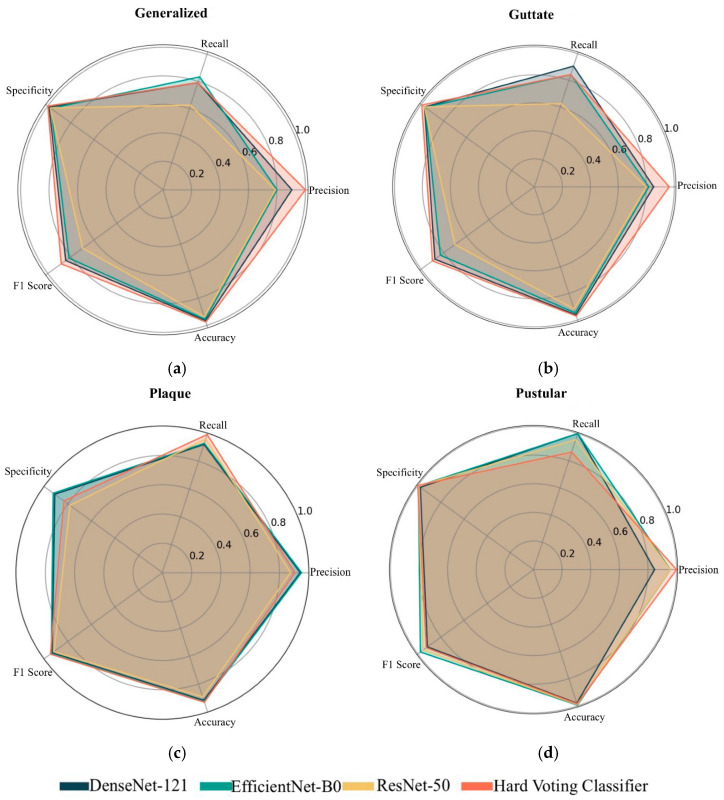
Comparison of performance metrics for each class across models: (**a**) generalized class, (**b**) guttate class, (**c**) plaque class, (**d**) pustular class.

The proposed HVC model achieved superior performance compared to individual TL models, with metrics including 93.14% accuracy, 96.75% precision, 87.28% recall, 91.44 F1 score, and 95.69% specificity. The performance metrics for each class in the dataset are also shown through the radar chart in Figure 11. Upon examining Figure 11, it is observed that the highest performance across all classes was achieved by the HVC model. While DenseNet-121 and EfficientNet-B0 delivered balanced results, they fell short of the performance exhibited by HVC. The Res-Net-50 model, on the other hand, struggled particularly in detecting positive examples. The most successful class for the models was the pustular class, with the HVC model achieving 100% recall, 100% specificity, and 92.68 F1 score. The guttate and plaque classes exhibited balanced performance across all models. The lowest performance was observed in the generalized class.

## 6. Discussions

This study presents a hybrid approach that combines TL and EL techniques for classifying psoriasis subtypes. The proposed model surpasses the performance of individual TL models by leveraging the feature extraction capabilities of TL on limited datasets and the decision making power of EL.

DenseNet-121 and EfficientNet-B0 exhibited balanced performance across all metrics; whereas, ResNet-50 struggled, particularly in detecting positive samples. In contrast, the HVC model achieved the highest precision, recall, and F1 scores, outperforming individual models. These results demonstrate the effectiveness of integrating diverse learning models in addressing complex and imbalanced datasets, such as those involving psoriasis subtypes.

Comparing with the literature given in Table 5, we conclude the following:

- This study differs from the literature, which predominantly relies on ready-made datasets, like DermNet, by utilizing a clinically rich, custom dataset including rare subtypes;
- A meticulous pre-processing procedure ensures high data quality and improved generalization;
- The multi-TL strategy combines DenseNet-121, EfficientNet-B0, and ResNet-50 models with HVC, achieving superior performance in classifying both rare subtypes and broader categories;
- The dataset’s compatibility with smartphone-captured images enhances the model’s practicality for real-world applications;
- This study focused on the deep learning-based classification of psoriasis images collected from a hospital outpatient clinic, with an emphasis on diagnostic assistance for dermatologists. Special forms of psoriasis, such as those affecting the scalp, nails, and fingers, were excluded from the analysis, though these cases were considered during the dataset labeling process, which resulted in a slight reduction in classification accuracy. Despite challenges, including the use of phone camera images that led to some classification errors, the study achieved promising results compared to the existing literature. The findings offer significant support to healthcare professionals, particularly in settings without access to advanced diagnostic tools like videodermatoscopy, making this approach valuable for rapid diagnosis in such environments. Future research aims to develop algorithms that address these specific psoriasis forms to further enhance diagnostic accuracy.

## 7. Conclusions

This study introduces a hybrid approach that integrates the strengths of DenseNet-121, EfficientNet-B0, and ResNet-50 TL models with the HVC for classifying psoriasis subtypes. The proposed model achieved superior performance compared to individual TL models, with metrics including 93.14% accuracy, 96.75% precision, 87.28% recall, 91.44 F1 score, and 95.69% specificity. Notably, the model achieved 100% precision and 100% specificity for the rare pustular class, making a significant contribution to the literature.

This work not only provides a scalable hybrid framework for analyzing other dermatological diseases but also demonstrates the effective use of smartphone-captured datasets, offering practical insights for similar studies. Given the chronic nature of psoriasis and its impact on healthcare systems, leveraging accessible devices like smartphones for high-performance predictions has the potential to reduce diagnostic burdens and improve patient care, particularly in resource-limited settings.

Future studies could focus on expanding the dataset, implementing more complex hybrid approaches, and testing the model’s integration into clinical and real-world scenarios for broader applicability.

## Figures and Tables

**Table 1:** Configuration of data preprocessing and augmentation techniques.

Category	Parameter	Detail	Value
Preprocessing	Gaussian Blur	Kernel Size	(5, 5)
	Thresholding	Value	45
	Thresholding	Max Intensity	255
	Morphological Erosion	Iterations	2
	Morphological Dilation	Iterations	2
	Contour Detection	Method	cv2.RETR_EXTERNAL, cv2.CHAIN_APPROX_SIMPLE
	Cropping Boundaries	Based on Extremes	Top, Bottom, Left, Right
Data Augmentation	Rotation		30
	Width Shift		0.2
	Height Shift		0.2
	Shear		0.2
	Zoom		0.2
	Horizontal Flip		True
	Fill Mode		Nearest

**Table 2:** Parameters and settings of callback functions.

Callback Function	Parameter	Value
Checkpoint	Monitor	val_accuracy
	Save Best Only	True
	Mode	auto
Early stopping	Monitor	val_accuracy
	Patience	5
	Mode	auto
Reduce LR	Monitor	val_accuracy
	Factor	0.3
	Patience	2
	Min Delta	0.001
	LR Mode	auto

**Table 3:** Performance metrics for each class across models without data augmentation.

Model	Class	Precision (%)	Recall (%)	Specificity (%)	F1 Score (%)
DenseNet-121	Generalized	86.67	54.17	98.78	66.67
	Guttate	94.74	56.25	99.36	70.59
	Plaque	81.17	99.21	53.23	89.29
	Pustular	0	0	100	0
	Average	65.645	52.4075	87.8425	56.6375
EfficientNet-B0	Generalized	74.07	83.33	95.73	78.43
	Guttate	86.67	81.25	97.44	83.87
	Plaque	89.76	90.48	79.03	90.12
	Pustular	50	33.33	98.9	40
	Average	75.125	72.0975	92.775	73.105
ResNet-50	Generalized	84.21	66.67	98.17	74.42
	Guttate	83.33	78.12	96.79	80.65
	Plaque	87.05	96.03	70.97	91.32
	Pustular	0	0	100	0
	Average	63.6475	60.205	91.4825	61.5975
HVC	Generalized	94.44	70.83	99.39	80.95
	Guttate	82.86	90.62	96.15	86.57
	Plaque	90.98	96.03	80.65	93.44
	Pustular	100	33.33	100	50
	Average	92.07	72.7025	94.0475	77.74

**Table 4:** Performance metrics for each class across models.

Model	Class	Precision (%)	Recall (%)	Specificity (%)	F1 Score (%)
DenseNet-121	Generalized	90.48	79.17	98.89	84.44
	Guttate	85.29	90.63	97.09	87.88
	Plaque	94.31	92.06	91.03	93.17
	Pustular	84.6	100	97.8	91.67
EfficientNet-B0	Generalized	80	83.33	97.22	81.63
	Guttate	81.82	84.38	96.51	83.08
	Plaque	95.12	92.86	92.31	93.9
	Pustular	95.65	100	99.45	97.78
ResNet-50	Generalized	78.95	62.5	97.78	69.77
	Guttate	80	62.5	97.09	70.18
	Plaque	87.68	96.03	78.21	91.67
	Pustular	95.45	95.45	99.45	95.45
HVC	Generalized	100	79.17	100	88.37
	Guttate	96.43	84.38	99.42	90
	Plaque	90.58	99.21	83.33	94.7
	Pustular	100	86.36	100	92.68

**Table 5:** Summary of studies.

Study	Methodology	Dataset	Results	**Contributions**
Syu et al. (2018) [8]	DNN-based CAD system for psoriasis detection	5700 images of normal and psoriatic skin	Test accuracy: 91%; training accuracy: 95%+	High-accuracy psoriasis detection using non-invasive DNN-based methods
Pal et al. (2018) [9]	DCNN and FCN for segmentation of psoriasis skin biopsy images	90 annotated biopsy images	RCPC: 88.01% (test); JC for dermis: 83.97%	Introduced an annotated dataset; emphasized FCN efficiency for segmentation tasks
Amin and Farooq (2021) [10]	ResNeXt101, ResNet-50, InceptionV3, DenseNet-121 for psoriasis detection	815 images (psoriasis, normal, other diseases)	Accuracy: 94% (binary); 79% (multiclass)	High-accuracy TL framework for psoriasis and similar skin diseases
Aijaz et al. (2022) [11]	CNN and LSTM-based DL for classifying psoriasis subtypes and normal skin	301 images (DermNet) + 172 BFU NTU images	CNN: 84.2%; LSTM: 72.3%	Proposed multiclass classification with detailed preprocessing and validation
Goswami et al. (2023) [12]	TL-based CNN classification using GoogleNet, SqueezeNet, and ResNet-18	306 images from DermNet	ResNet-18 accuracy: 93.62%	Effective psoriasis subtype classification using TL and MATLAB (accessed on 2 August 2023, from https://www.mathworks.com)
Arunkumar and Jayanna (2023) [13]	Depth-wise CNN with MobileNet for severity estimation	5951 images (balanced across severity levels)	Test accuracy: 90%; F1 score: 0.94	Lightweight MobileNet for handheld devices, dermatologist-validated
Yaseliani et al. (2024) [14]	Ensemble CNN (ResNet) with MCDM for classification and treatment	2100 RGB images (DermNet)	Binary: 91.90%; multiclass: 93.29%	Integrated classification with treatment recommendation framework
Nieniewski et al. (2023) [15]	VGG16/VGG19 feature extraction + SVM with majority voting	3570 images (smartphone)	Accuracy: 80.08%; recall: 85.33%	Smartphone-based CAD for psoriasis vs. other dermatoses
Moon et al. (2023) [16]	MS-DAM integrated with EfficientNet for severity classification	44 Korean patients; 5 severity levels	F1 score: 0.93	Improved severity classification with MS-DAM
**This Study (2024)**	Hybrid model with multi-TL (DenseNet-121, EfficientNet-B0, ResNet-50) and HVC	930 images from Fırat University Hospital Dermatology Clinic	Accuracy: 93.14%; precision: 96.75%; F1 score: 91.44%	Hybrid TL-EL model with high performance in rare subtypes for real-world use

## Data Availability

The data presented in this study are available on request from the corresponding author. The dataset used in this study was obtained by having the necessary ethical permissions from Faculty of Medicine, Fırat University.
